# Comparative Analysis of Two Types of Combined Power-Over-Fiber and Radio-Over-Fiber Systems Using Raman Amplification for Different Link Lengths †

**DOI:** 10.3390/s25134159

**Published:** 2025-07-04

**Authors:** Paulo Kiohara, Romildo H. Souza, Véronique Quintard, Mikael Guegan, Laura Ghisa, André Pérennou, Olympio L. Coutinho

**Affiliations:** 1 Laboratório de Guerra Eletrônica, Instituto Tecnológico de Aeronáutica (ITA), São José dos Campos 12228-900, SP, Brazil; romildo@ita.br (R.H.S.); olympio@ita.br (O.L.C.); 2 Laboratoire des Sciences et Technologies de l’Information, de la Communication et de la Connaissance (UMR 6285 Lab-Sticc), École Nationale d’Ingénieurs de Brest (ENIB), Centre National de la Recherche Scientifique (CNRS), 29238 Brest, France; quintard@enib.fr (V.Q.); guegan@enib.fr (M.G.); telescu@enib.fr (L.G.)

**Keywords:** radio over fiber, power over fiber, Raman amplification, remote sensors

## Abstract

The use of analog radio-over-fiber (RoF) systems combined with power-over-fiber (PoF) systems has been proposed in recent years for applications involving remote sensors used in hazardous environments or where electrical wiring may be impractical. This article presents a hybrid architecture topology that combines PoF and RoF, using Raman amplification to obtain RF gain. The first emphasis is placed on the use of two types of high-power laser sources (HPLSs) for the PoF system: a 1480 nm Raman-based HPLS and a 1550 nm HPLS that is based on an erbium-doped fiber amplifier (EDFA). The second emphasis of this paper is on how these two HPLSs simulate Raman scattering (SRS) in the fiber, considering different lengths of SMF 28 for the link. Thus, a comparative analysis is proposed considering the effects induced on the RF signal, mainly focused on its RF power gain ($G_{RF}$), noise figure (NF), and spurious-free dynamic range (SFDR). The obtained results show that the architecture using a PoF system based on the 1550 nm HPLS benefits from a lower noise figure degradation, even when the noise generated by the optical amplification is considered.

## 1. Introduction

Over the past few years, the telecommunication industry has experienced a huge rise, resulting in an increased demand for the number of remote antenna units available, greater reliability, and coverage over longer distances. To meet these requirements, several technologies have been studied and developed, including radio-over-fiber (RoF), power-over-fiber (PoF), and optical amplification, such as Raman amplification, to increase the signal power level to reach long distances profitably [1,2,3,4,5].

The combination of all these techniques has been proposed as a fully photonic system to achieve high-speed data transmission on long-distance fronthaul and sensor systems. Studies have been carried out to evaluate the crosstalk between the data signal and the HPLS, evaluating the error vector magnitude (EVM) and bit error rate (BER) for Raman links using multimode fibers (MMFs) [6]. Additionally, research on Raman amplification for passive optical networks (PONs) using different fiber lengths vs. BER [7] was recently published. The influence of Raman amplification on the 5G signal through the EVM has also been studied [3]. An approach considering the use of PoF and RoF on the same optical fiber has been presented, but without considering Raman amplification [8].

The versatility of these techniques extends to numerous applications, including powering remote sensors [9], monitoring systems [10,11], remote antenna units [12], IoT devices [12,13,14,15], and satellite antennas [16]. A notable application for low-power systems is the implementation of drone control networks. Drones have recently been employed in various fields, ranging from video capture to synthetic aperture radar (SAR) operations, among others [17,18]. Another promising use case is the deployment of antenna networks for drone detection and tracking [19].

Several studies have evaluated the performance of RoF-PoF systems with Raman amplification in terms of EVM or BER [3,6,7,12] for specific modulation formats such as QPSK, 16QAM, 64QAM, and 256QAM. This study presents a complementary analysis of analog figures of merit for a bandwidth of 2 MHz, regardless of the data modulation format, offering an alternative to traditional digital transmission analyses, which are typically evaluated through EVM or BER. The evaluation focuses on three key parameters: RF gain ($G_{RF}$), noise figure (NF), and spurious-free dynamic range (SFDR). The evaluation provides a comprehensive assessment of the system’s performance prior to data processing.

In a previous publication, we presented a study of the effects of Raman amplification on RoF link performance by using classical figures of merit [20], but without considering the influence of the link length. However, the length of the used fiber can have a significant effect on noise during transmission, given the interactions between the high power and the RF optical carrier. In particular, amplified spontaneous emission (ASE) noise and relative intensity noise (RIN) transfer can be generated during propagation along the fiber. Additionally, these interactions can have different significant effects on RoF performance depending on the type of high-power laser source (HPLS) used for the PoF system.

The main objective of this paper is to present an optical and RF experimental analysis of a hybrid combined PoF-RoF architecture using Raman amplification within the context of two types of PoF systems. To achieve high power (up to 5 W) and stimulate the Raman scattering, we use two common high-power sources: an Erbium-Doped Fiber Amplifier (EDFA) associated with a 1550 nm white source laser and a Distributed Raman Laser. The proposed comparative analyses of the experimental and theoretical results focused on the RF signal degradation caused by the noise generated due to Raman optical amplification. A comparative study is also performed for different lengths of the SMF link (5 km, 10 km, and 15 km) to consider the influence of the link length.

## 2. RoF and PoF Receiving Analog RF Link

**Figure 1:**
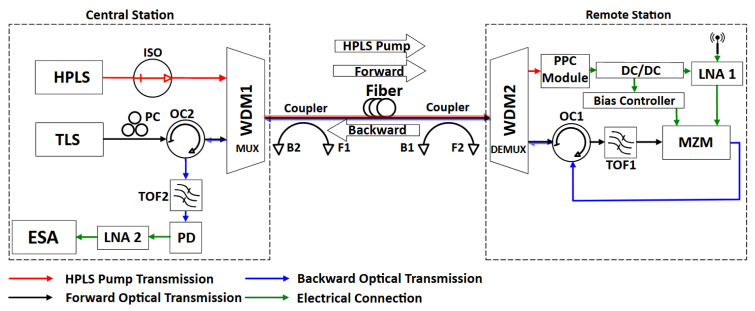
Hybrid RoF-PoF link for receiving antenna (Rx).

Fronthaul links can be used to transmit both analog and digital signals from a receiving antenna or to a transmitting antenna, connecting a remote station with a central station [20]. The hybrid architecture proposed in this work for the link shown in Figure 1 works as a receiving system.

In the central station, the HPLS provides optical power, and the tunable laser source (TLS) provides the RoF optical carrier. An isolator (ISO) is used to protect the HPLS from back-reflected signals. The optical circulator (OC2), located in the central station, allows for the transmission of the RoF optical carrier to the remote station in the forward direction. The two optical signals from HPLS and TLS are combined in the wavelength division multiplexer (WDM1) and transmitted over a single mode SMF-28. Both the HPLS and TLS optical signals are received by the WDM2. Simultaneously, the optical coupler OC2 receives the backward RoF signal from the remote station, coupling it to the photodetector (PD).

In the remote station, the Mach–Zehnder modulator (MZM) modulates the optical carrier emitted in the central station with the RF signal received by the antenna after being preamplified by the LNA1. The modulated signal is then transmitted back in the backward direction via an optical circulator (OC1). Tunable optical filters are used to minimize optical noise effects at the PD input (TOF2) in the central station as well as at the MZM input (TOF1) in the remote station. A polarization controller (PC) is used to remotely control the optical polarization at the input of the MZM.

The HPLS signal received by the WDM2 is directed to the 1 × 8 optical splitter with a photovoltaic power converter (PPC) module with eight PPCs. The optical energy is then converted into electrical energy to supply electronic devices such as LNA1, the DC/DC converter, and the MZM’s Vbias Controller. The Vbias controller is responsible for keeping the MZM at its quadrature operating point and is essential to the operation of the system.

As a bidirectional system, the Raman gain provided by stimulated Raman scattering (SRS) can be observed in both the forward and backward propagation directions. However, the SRS not only amplifies the power of the transmitted signal but also generates and amplifies noise, which can degrade the overall system performance [5]. For experimental purposes, two optical couplers are employed to estimate the optical gains in both the forward transmission direction and the backward transmission direction of the system. In the forward direction, the couplers are positioned at the input (F1) and output (F2) of the optical fiber, whereas in the backward direction, they are placed at the input (B1) and output (B2).

The first PoF system uses a 1480 nm Raman-based HPLS. In this case, the TLS wavelength is set at 1550 nm, corresponding to an optimal wavelength spacing of 70 nm to benefit from a good compromise between gain and noise [20]. The second PoF system uses a 1550 nm HPLS based on an EDFA. In this case, the TLS wavelength is set at 1610 nm, corresponding to a wavelength spacing of 60 nm, which is not optimal due to limitations in our setup. This results in a lower amplification capacity for the EDFA-based system [9].

## 3. Experimental Measurement Procedure and Results

The performance of the RoF system is analyzed using the standard RF figures of merit. As the link undergoes a process of Raman optical amplification, it is also essential to consider the influence of this amplification on the system’s performance.

### 3.1. Experimental Measurement Procedure

- RF Power Gain Measurement

Take as a reference Figure 1. The RoF RF gain, $G_{RF},$ is measured as the ratio between the RF power at the LNA2 output, $p_{RFout}$, and the available RF power at the LNA1 input, $p_{RFin}$. It can be expressed in decibels (dB) by [2]:

(1) $$G_{RF}^{M}=P_{RFout}-P_{RFin}.$$

- Noise Figure Measurement

The NF can be measured as the ratio between the output noise power spectral density (PSD) of the system, $n_{out}$, and the noise PSD at the output only due to the system input noise power, $n_{in}$. In dB, the measured NF is expressed by the following formula [2]:

(2) $${NF}^{M}=N_{out}-{(N}_{in}+G_{RF}),$$

where $N_{in}$ is the system input thermal noise at the reference temperature, which is considered to be −174 $dBm.{Hz}^{-1}$ [2].

- Spurious-free dynamic range measurement

The third-order SFDR is a measurement of the dynamic range between the minimal RF output power and the maximum RF output power that is free from the third-order intermodulation signal. It is measured via a two-tone experiment with a Δf ± 2 kHz and can be expressed in ${dB.Hz}^{2/3}$ as follows [2]:

(3) $${SFDR}^{M}=\frac{2}{3}\left({IIP}_{3}-NF+N_{in}\right),$$

where ${IIP}_{3}$ is the input third-order intercept point.

### 3.2. Theoretical Procedure

- RF Power Gain Theory

The theoretical RF gain for an optical fiber link using an intensity modulator and direct detection (IM/DD), which takes into account the optical amplification of the signal through the stimulated Raman scattering, is defined in dB as [2]:

(4) $$G_{RF}^{T}=20\log\left(\frac{Rg_{op}p_{L}l_{op}\pi Z}{2V_{\pi }}\right),$$

where R is the photodetector’s responsivity, $g_{op}$ is the link optical gain, $p_{L}$ is the TLS optical power, $l_{op}$ is the link to optical loss, $V_{\pi }$ is the half-wave voltage, and Z is the common impedance of the link.

- Noise Figure Theory

Typically, four noise components are considered in back-to-back RoF links. These are PSD thermal noises from the MZM ($n_{th}^{MZM})$ and PD ($n_{th}^{PD})$ impedances, the PSD relative intensity noise (RIN) of the TLS ($n_{rin}^{TLS})$ and the PSD shot noise ($n_{shot}^{TLS}$) [2]. However, Raman optical amplification introduces new noise components into the system.

During the optical amplification process, the relative intensity noise (RIN) from the HPLS can be transferred to the optical signal of the TLS. This crosstalk arises from the nonlinear interaction between the signals of the HPLS and the TLS. This phenomenon is commonly referred to as RIN transfer and occurs during forward transmission ($n_{Tf}^{rin}$) and backward transmission ($n_{Tb}^{rin}$) [5,20].

The use of square-law photodetectors requires careful consideration, as their output is proportional to the square of the amplitude of the incident-modulated optical field. This nonlinearity leads to beat noise, which arises from the interference of optical waves with slightly different frequencies or phases. When converted to the electrical domain, this interference generates noise that can degrade the quality of the received signal. In the case of the proposed link, these beats are the beats between the ASE-TLS ($n_{TLS}^{ASE}$) and the ASE-ASE ($n_{ASE}^{ASE}$) in the PD [5].

Finally, one last noise to consider is the PSD ASE shot noise ($n_{shot}^{ASE}$). Equation (5) shows the theoretical NF (${NF}^{T})$ for a Raman amplified link [2,5].

(5) $${NF}^{T}=10\log\left(1+\frac{n_{th}^{MZM}+n_{th}^{PD}+n_{shot}^{TLS}+n_{shot}^{ASE}+n_{rin}^{TLS}+n_{TLS}^{ASE}+n_{ASE}^{ASE}+n_{Tf}^{rin}+n_{Tb}^{rin}}{g_{RF}^{T}k_{B}T_{s}}\right),$$

where $k_{B}$ is the Boltzmann constant and where $T_{s}$ is the standard room temperature in Kelvin.

- Spurious-free dynamic range theory

The theoretical SFDR (${SFDR}^{T}$) is defined by Equation (6) [2]:

(6) $${SFDR}^{T}={10\log\left[\frac{4V_{\pi }^{2}}{\pi^{2}Zk_{B}T_{s}{NF}^{T}}\right]}^{2/3}$$

In this case, there are no changes to the definition of the ${SFDR}^{T}$ for a system with Raman amplification, but the new definition of the ${NF}^{T}$ to the system is also taken into account in Equation (6).

## 4. Optical and Radio Frequency Measurement Analysis

### Optical Measurements Analysis

The experiments were carried out using three different fiber lengths: 5 km, 10 km, and 15 km. All the measurements were performed with a fixed TLS optical power of 0 dBm and with the HPLS optical power starting at 30 dBm.

- Raman amplification

**Figure 2:**
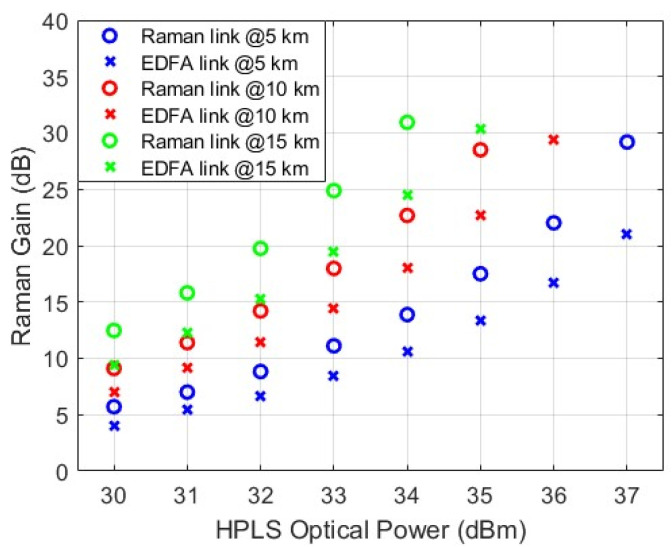
Experimental Raman gains for 5 km, 10 km, and 15 km SMF-28.

The total Raman gains of the links obtained via Raman-based and EDFA-based HPLS are shown in Figure 2, and they are the sums (in dB) of the forward and backward gains. The forward gains were measured by taking the optical power measured from points F1 and F2, and the backward gains were measured from points B1 and B2 (Figure 1).

The Raman-based HPLS revealed that the measured maximum Raman gains for fiber lengths of 5 km, 10 km, and 15 km were 29.1 dB, 28.5 dB, and 30.9 dB, respectively. For the EDFA-based HPLS, the maximum gains were 21 dB, 29.7 dB, and 31.2 dB, respectively. As expected, theoretically, the Raman gain is greater for longer fibers [4]. Therefore, with long fibers, less input HPLS power is needed to achieve similar optical gain values [5].

However, in both HPLS setups, a limitation is observed in the amount of HPLS optical power that can be transmitted over lengths of 10 km and 15 km. For the link with the Raman-based HPLS, the limits are 35.6 dBm and 34.3 dBm for lengths of 10 km and 15 km, respectively. For the link with the EDFA-based HPLS, the limits are 36.4 dBm and 35.2 dBm, respectively. Increasing the HPLS optical power above these levels causes instabilities in the optical power output of the link, followed by a huge drop in the Raman amplification and the availability of optical power on the PPC at the remote station. These effects are associated with HPLS pump depletion, a phenomenon in which a substantial portion of the optical energy is transferred to the Stokes wave [5,21]. In the depletion regime, nonlinear interactions between the pump and Stokes waves become more pronounced, potentially causing instabilities. If the HPLS pump fails to sustain a stable energy transfer to the Stokes wave, fluctuations and irregularities may arise. Consequently, this leads to time-dependent variations in both the TLS signal and the entire ASE spectrum, which cannot maintain a constant optical power [5,21].

**Figure 3:**
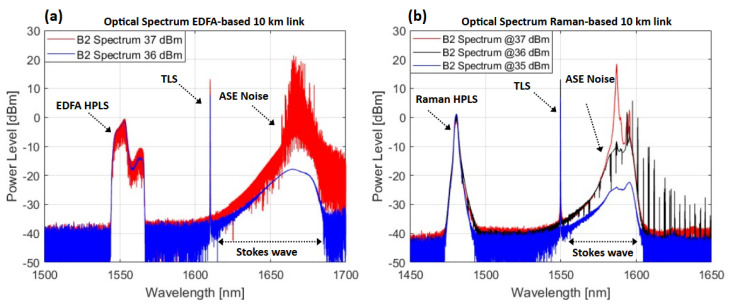
Optical spectrum for the measured point B2 for (**a**) 10 km of SMF−28 for the EDFA−based link at an input HPLS of 36 dBm and 37 dBm; (**b**) 10 km of SMF−28 for the Raman-based link at an input HPLS of 35 dBm, 36 dBm and 37 dBm.

As an example, Figure 3 shows the optical spectrum measurements at the output point B2 of the optical fiber shown in Figure 1.

The spurious peaks observed in both the EDFA-based link (Figure 3a) and the Raman-based link (Figure 3b) degrade the optical and RF performance, causing fluctuations in the Raman optical gain and the RF metrics. For this reason, the limits presented above are used for the different lengths and types of HPLS.

- Power over Fiber Analysis

**Figure 4:**
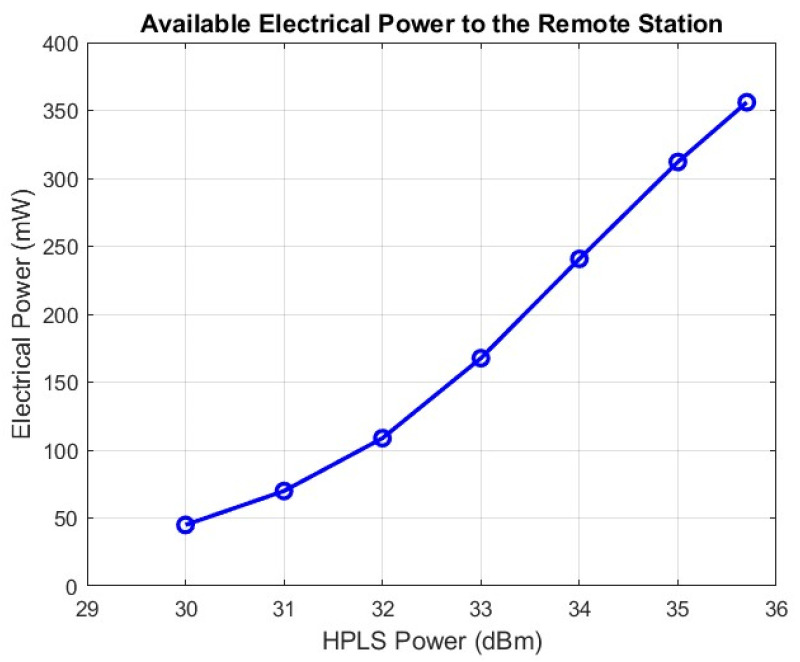
Electrical power available at the output of the PPC module at the remote station for a 5 km fiber length.

Considering the Vbias controller and LNA1, the electrical power required to supply electronic devices is approximately 263 mW. An experiment to validate the energy system was carried out with a 5 km Raman-based link. With a 1 × 8 optical splitter in the PPC module and an input power limit of 190 mW per PPC, a conversion efficiency of approximately 22% was achieved. As a result, with an HPLS optical power of 35.7 dBm, which guarantees safe operation of PPCs, the PPC module produced a maximum electrical output of 356 mW. Figure 4 shows the measured electrical power at the output of the PPC module (8_PPCs) for a 5 km fiber length.

The DC/DC converter is used to match the voltage and current required by the Vbias controller and the LNA1. Its minimum efficiency is 90%, which results in approximately 320 mW of electrical power. More than enough to keep the system running. Similar results can be obtained for the EDFA-based link since the conversion efficiency of the PPC is similar for the 1550 nm wavelength.

**Figure 5:**
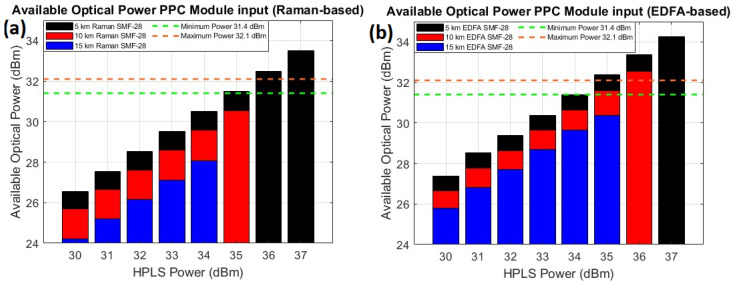
Optical power available at the input of the PPC module at the remote station (**a**,**b**).

Figure 5 illustrates the optical power available at the input of the PPC module at the remote station (Figure 1) for different fiber lengths and for the Raman-based link (Figure 5a) and the EDFA-based link (Figure 5b).

The orange horizontal lines represent the maximum input optical power to respect the safety limit of the PPCs, which is required to obtain 356 mW of electrical power at the output of the PPC module. The green horizontal line represents the minimum input optical power required to meet the 263 mW electrical power threshold necessary for the proper operation of the remote station’s electronic components.

For the Raman-based link, only the 5 km fiber length satisfies both the minimum and maximum power requirements at the PPC module input. In contrast, for the EDFA-based link, fiber lengths of 5 km and 10 km meet these power constraints. Since these links can achieve an input power above the maximum optical power of 32.1 dBm, the PPC module can be reconfigured with a 1 × 16 optical splitter to enhance optical power reception at the Remote Station.

- Radio frequency measurement analysis

**Figure 6:**
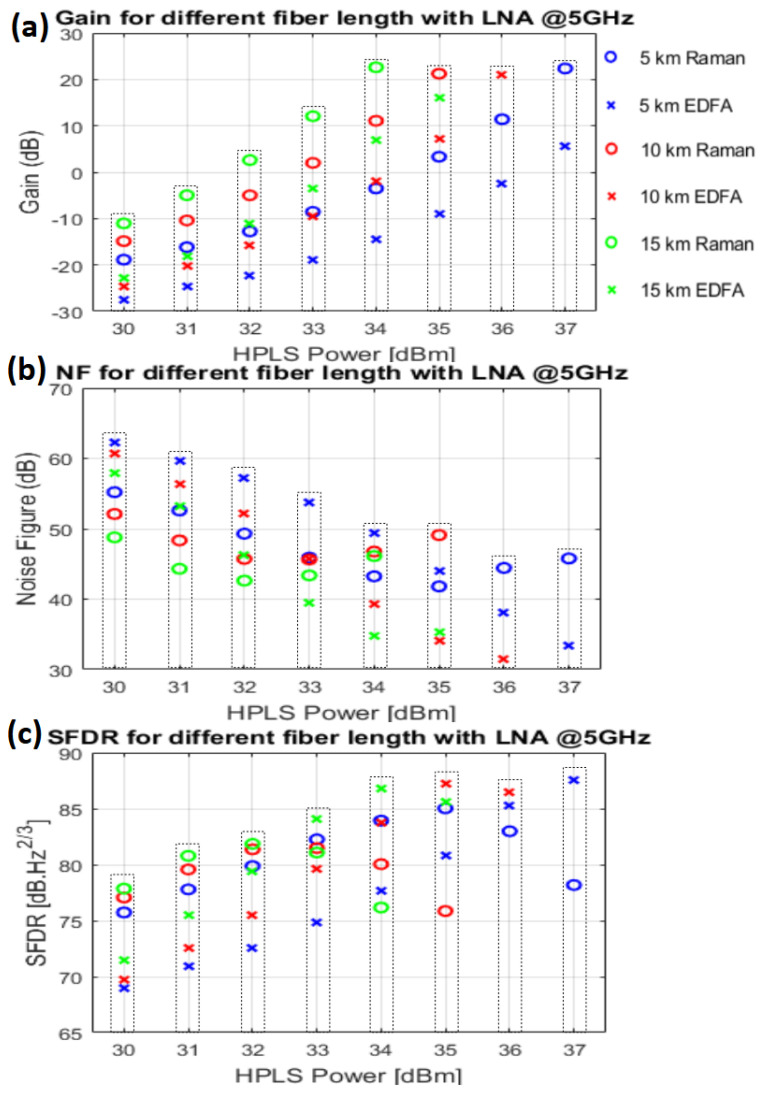
Measured RF gain, noise figure, and SFDR for the Raman-based and EDFA-based setups.

The RF measurements were conducted using a 5 GHz RF signal with a continuous wave (CW). The LNA1 has a gain of 24 dB with a NF of 1.9 dB, connected to the MZM input with a Vπ of 5.2 V. The gain of the LNA2 is 35 dB, with a NF of 1 dB, connected after the PD with a responsivity of 0.65 A/W. An RF generator is used instead of the antenna to feed the MZM RF input with a power of 0 dBm. Figure 6 shows the RF gain, NF, and SFDR measurement results of the links with Raman-based and EDFA-based HPLS, considering the three fiber lengths.

Figure 6a presents the measured RF gain at various HPLS power levels, calculated using Equation (1). The RF gain is directly related to the Raman gain behavior shown in Figure 2. The maximum gain achieved was 22.6 dB for a fiber length of 15 km with a Raman-based HPLS and an input power of 34 dBm.

However, high gains do not necessarily indicate good NF values. As the length of the fiber and the HPLS power increase, optical amplification due to stimulated Raman scattering can produce high levels of ASE noise and RIN transfer, which degrades the NF of the system. By applying Equation (2), it is possible to define the measured noise figure (Figure 6b). For the Raman-based HPLS and for lengths of 5 km, 10 km, and 15 km, the NF starts to increase from the input HPLS powers of 35 dBm, 33 dBm, and 32 dBm, respectively, although the RF gain continues to increase. The same effect can be observed for the EDFA-based HPLS, with lengths of 10 km and 15 km.

Through Equation (3), it is possible to define the measured SFDR (Figure 6c). The SFDR begins to decrease at the same HPLS power level as the NF increases for different fiber lengths because of the strong relationship between the SFDR and NF, as shown in Equation (3).

**Figure 7:**
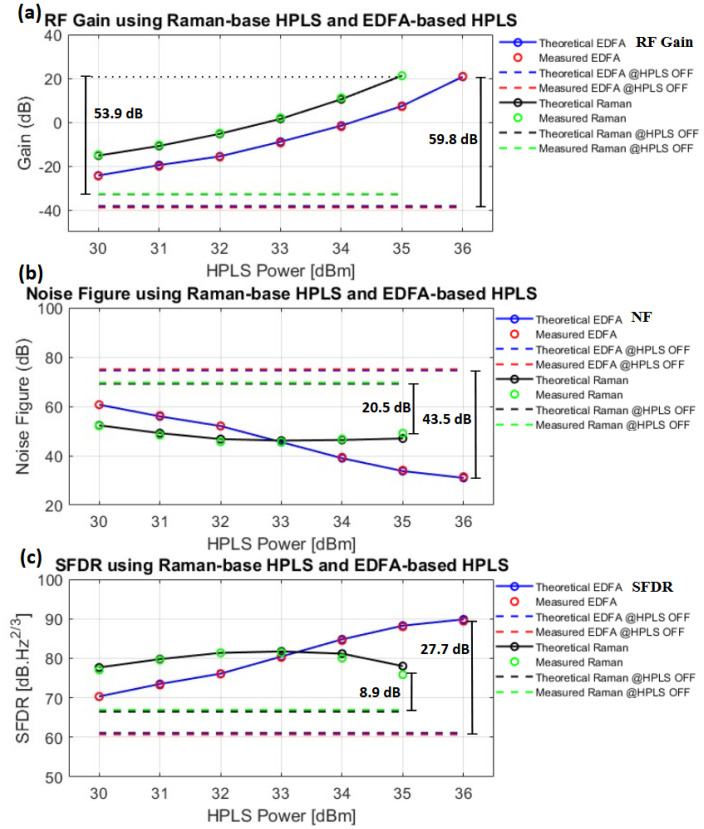
Theoretical and measured RF metrics for the 10 km Raman-based and EDFA-based links for 5 GHz: (**a**) RF gain; (**b**) noise figure; (**c**) spurious-free dynamic range.

The RF results presented in Figure 6 are validated by comparing the experimental and theoretical results. Using Equations (4)–(6), it is possible to calculate $G_{RF}^{T}$, ${NF}^{T}$, and ${SFDR}^{T}$, which are shown in Figure 7 for the 10 km Raman-based and EDFA-based links.

In Figure 7a–c, the green dots and black lines represent the experimental and theoretical results, respectively, for the Raman-based link. Similarly, the red dots and blue lines represent the experimental and theoretical results of the EDFA-based link. The dashed lines in the respective colors represent the experimental and theoretical results when the HPLS is OFF, i.e., when the system is not under the effect of Raman optical amplification.

For the RF gain (Figure 7a), there is a good match between theory and experiment. The maximum $G_{RF}^{M}$ for the Raman-based link is 21.3 dB at an input HPLS power of 35 dBm, whereas for the EDFA-based link, the $G_{RF}^{M}$ is 20.9 dB at an input HPLS power of 36 dBm.

Figure 7b shows the measured and theoretical NF. For the Raman-based link, ${NF}^{M}$ is 49.1 dB at an input HPLS power of 35 dBm, whereas for the EDFA-based link, ${NF}^{M}$ is 31 dB at an input HPLS power of 36 dBm. For the Raman-based link, a noticeable increase in the NF occurs between 33 dBm and 35 dBm. This rise is attributed to the noise components $n_{Tf}^{rin}$ and $n_{Tb}^{rin}$, whose influence becomes more significant at high optical power levels [9]. In the EDFA-based link, the increase in NF is less intense due to the low RIN of the HPLS ($n_{rin}^{HPLS})$ used in the link [2,9]. Between ${NF}^{M}$ and ${NF}^{T}$, there is a mismatch of 2 dB for a power of 35 dBm on the Raman-based link. This difference is not observed for noise when Equation (5) is applied to a backward and forward link separately, as is usually the case in the literature [9,11], and has no influence at low noise levels, as seen for the EDFA-based link, which has a good match between the curves.

Figure 7c presents the measured and theoretical ${SFDR}^{M}$. For the Raman-based link, the theoretical SFDR is 75.8 ${dB.Hz}^{2/3}$ at an input HPLS power of 35 dBm, whereas for the EDFA-based link, it is 89.4 ${dB.Hz}^{2/3}$ at an input HPLS power of 36 dBm. A discrepancy of approximately 2 dB between ${SFDR}^{M}$ and ${SFDR}^{T}$ for the Raman-based link at an input optical power of 35 dBm can be attributed to the mismatch in the NF. As indicated in Equation (6), the SFDR is directly influenced by the NF.

Despite a slight mismatch between the experimental and theoretical results at higher power levels, particularly for the Raman-based link, the overall agreement between the observed results was good.

**Figure 8:**
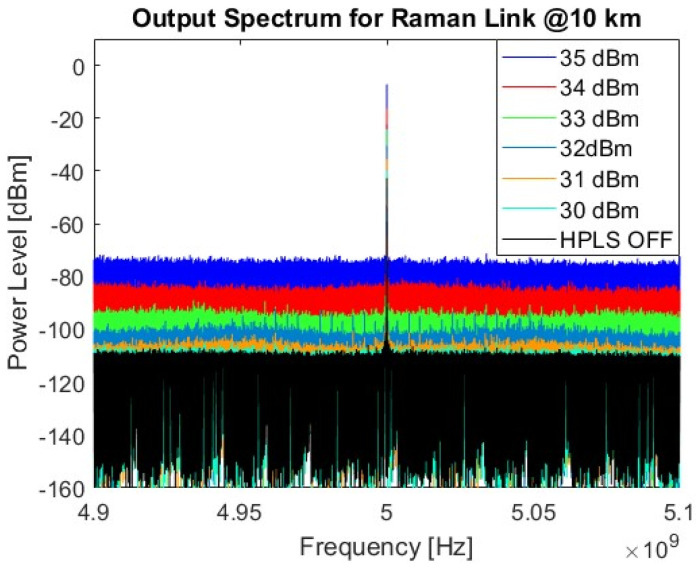
Electrical spectra of the Raman links for different HPLS powers.

To better visualize the noise impact generated by optical Raman amplification on the RoF system, the RF signal spectrum is measured at the LNA2 output for different HPLS power levels over the 10 km fiber length. Figure 8 shows the output RF spectrum for the Raman-based HPLS. An RF output power of −7.2 dBm was measured for an input HPLS power of 35 dBm, with a signal-to-noise ratio (SNR) of approximately 65 dB. A significant power increase of 37 dB in the noise floor was observed when the maximum HPLS power of 35 dBm was compared with the HPLS power off.

**Figure 9:**
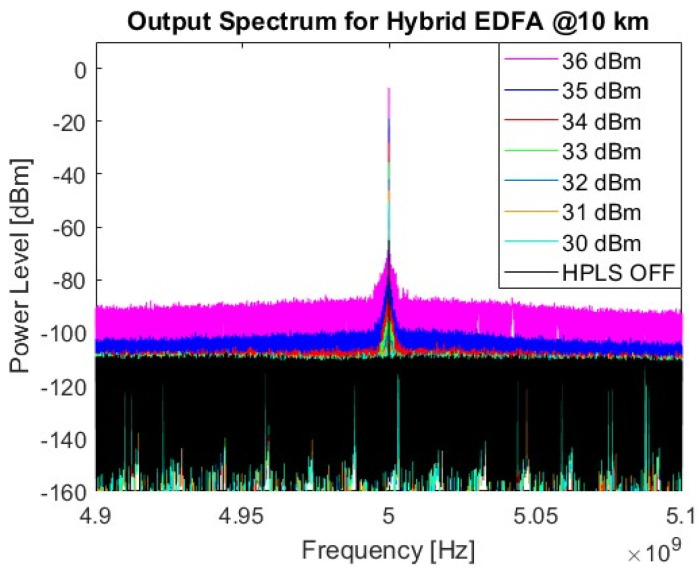
Electrical spectra of the EDFA link for different HPLS powers.

Figure 9 shows the measured output RF spectrum for the EDFA-based HPLS, with an RF signal power of −7.3 dBm and an SNR of approximately 81 dB for an input power of 36 dBm. The difference in noise floor power level between the HPLS off and the HPLS powered at 36 dBm is 20.5 dB.

The RoF noise floor output power difference between the two systems is 16.2 dB, considering the highest HPLS power for each PoF system. Although the EDFA-based link requires more power to reach the same RF signal level, the noise generated by the optical amplification is considerably lower.

Table 1 presents the RF metrics and the power provided at the PPC input ($P_{PPC}$) as a function of both HPLS and different link lengths, with a focus on the lowest measured ${NF}^{M}$.

The lowest measured NF for the EDFA-based HPLS case is 31 dB at an input HPLS power of 36 dBm for a fiber length of 10 km. For the Raman-based HPLS, the lowest NF is 41.8 dB at an input HPLS power of 35 dBm and for a fiber length of 5 km. A deeper analysis of the measurements in Figure 6 confirms that the EDFA-based HPLS outperforms the Raman-based HPLS in terms of NF for identical power levels or for equivalent RF gains. However, when the RF gain and NF are compared (Table 1), the EDFA-based HPLS yields a gain of 21.3 dB at its lowest NF value, whereas for the Raman HPLS, the gain at the lowest NF is only 3.3 dB. To obtain a comparable RF gain of 21 dB (Figure 6) with the Raman-based link, its input HPLS power must be increased to 37 dBm for 5 km fiber, resulting in a significant NF to 46 dB in this case.

Regarding the electrical power required at the remote station, both the 5 km Raman-based and EDFA-based HPLS configurations are viable options. Beyond these, only the 10 km EDFA-based configuration meets the minimum requirements. However, the 5 km Raman-based configuration exhibits higher NF levels, while the 5 km EDFA-based configuration has lower RF gain compared to the optimal values achieved by the 10 km EDFA-based HPLS. This suggests that the 10 km EDFA-based HPLS is also the most suitable for applications at distances of less than 10 km, offering a better trade-off between RF performances and power requirements for our application.

More generally, these results highlight a key trade-off: while the EDFA-based HPLS exhibits lower noise figure degradation, it does so at the expense of higher input HPLS power or longer fiber length. This increase in SMF length reduces the power received at the remote station, which impacts the overall efficiency of the PoF system, and limits the feasibility of using a new LNA with a better NF, as the latter will consume more electrical power.

Another limiting factor is the unconventional use of 1610 nm data transmission in the EDFA-based link, which may pose compatibility challenges for existing optical systems. Consequently, the Raman-based link presents a more practical alternative in scenarios where higher noise figure (NF) levels are not a critical constraint.

## 5. Conclusions

A hybrid PoF-RoF architecture topology using Raman has been proposed and studied, focusing on the use of two types of HPLS: a 1480 nm Raman-based HPLS and a 1550 nm EDFA-based HPLS. This study investigated the performance over different link lengths of 5 km, 10 km, and 15 km of SMF-28, offering a comparative analysis based on optical Raman gain and theoretical and experimental RF performance metrics. A strong correlation between the experimental and theoretical results validated the robustness of the proposed models.

The results highlighted a critical limitation on the maximum input HPLS power that can be transmitted through the fiber without causing instabilities for different lengths of fiber. These instabilities, influenced by the stimulated Raman scattering effect and high optical power levels, impose limitations on the PoF-RoF system in terms of optical and RF performances. Despite this limitation, optical Raman amplification demonstrated notable improvements in RF gain, NF, and SFDR.

The hybrid Raman-based and EDFA-based links achieved comparable RF gains of approximately 20 dB for fiber lengths of 10 km and 15 km. However, at 5 km, only the hybrid Raman-based link maintained this RF gain level due to the lower amplification at this length in the EDFA-based link. When compared with the EDFA-based link, performance degradation was observed at high input HPLS powers in the Raman-based configuration, particularly in NF and SFDR, making the link less suitable for scenarios requiring a low-noise operation.

Among the configurations and fiber lengths studied, the EDFA-based HPLS for the 10 km link emerged as the optimal choice, offering the lowest NF of 31 dB and an RF gain comparable to that of the best Raman-based configuration. Furthermore, the 10 km EDFA-based link showed approximately 11 dB less NF than the best-measured NF for the Raman-based link while maintaining sufficient optical power to meet the energy demands at the remote station and higher RF gain. The EDFA-based system’s consistent performance makes it suitable not only for the 10 km links but also for shorter distances, offering the best trade-off between optical and RF performance.

The PoF system successfully achieved its objectives by effectively powering the bias controller and the low-power LNA1 in the remote station. The operation of these electronic components required approximately 263 mW of electrical power, which was met using fiber lengths of 5 km in the hybrid Raman-based link, and 5 km and 10 km in the hybrid EDFA-based link.

Therefore, the system demonstrated satisfactory performance in both RoF and PoF, enabling remote operation as a fully photonic system that supports both data transmission and power delivery through a single SMF-28 fiber.

Future research should explore potential applications in more complex environments, such as WDM networks, where various HPLS can be employed to enhance signal amplification across multiple bands. Additionally, integrating a phase modulator could help reduce power consumption in the remote station, facilitating the implementation of a better LNA. Furthermore, while analog RF metrics offer a comprehensive perspective on link performance before analog-to-digital conversion, evaluating metrics such as EVM and BER is essential for assessing performance post-conversion. This would establish a clearer correlation between the performance of the analog RF characteristics and its digital performance.

## Figures and Tables

**Table 1:** Optical and RF metrics for the lowest NF in different setup configurations.

Fiber Length	5 km		10 km		15 km	
HPLS	Raman	EDFA	Raman	EDFA	Raman	EDFA
${NF}^{M}\;$(dB)	41.8	33.3	45.6	31	42.6	34.7
$G_{RF}^{M}$(dB)	3.3	5.5	2	21.3	2.6	8.8
${SFDR}^{M\;}$(${dB.Hz}^{2/3}$)	85	87.5	81.5	86.4	81.8	86.8
$P_{HPLS}\;$(dBm)	35	37	33	36	32	33.4
$P_{PPC}\;$(dBm)	31.5	34.3	28.4	32.6	26.2	29.6

## Data Availability

The data are contained within the article.

## Abbreviations

The following abbreviations are used in this manuscript:

ASE	Amplified Spontaneous Emission
BER	Bit Error Rate
OC1	Optical Circulator 1
OC2	Optical Circulator 2
EDFA	Erbium-Doped Fiber Amplifier
EVM	Error Vector Magnitude
HPLS	High Power Laser Source
ISO	Isolator
IM/DD	Intensity Modulation and Direct Detection
MMF	Multimode Fibers
MZM	Mach–Zehnder Modulator
NF	Noise Figure
PD	Photodetector
PPC	Photovoltaic Power Converter
PoF	Power over Fiber
PON	Passive Optical Networks
PSD	Power Spectral Density
RIN	Relative Intensity Noise
RoF	Radio over Fiber
SMF	Single-mode fiber
SFDR	Spurious-Free Dynamic Range
SRS	Stimulate Raman Scattering
TLS	Tunable Laser Source
TOF1	Tunable Optical Filter 1
TOF2	Tunable Optical Filters 2
WDM1	Wavelength Division Multiplexer 1
WDM2	Wavelength Division Multiplexer 2
